# Soluble factors from stellate cells induce pancreatic cancer cell proliferation *via* Nrf2-activated metabolic reprogramming and ROS detoxification

**DOI:** 10.18632/oncotarget.9165

**Published:** 2016-05-04

**Authors:** Yuan Seng Wu, Chung Yeng Looi, Kavita S. Subramaniam, Atsushi Masamune, Ivy Chung

**Affiliations:** ^1^ Department of Pharmacology, Faculty of Medicine, University of Malaya, Kuala Lumpur, 50603 Malaysia; ^2^ Division of Gastroenterology, Tohoku University Graduate School of Medicine, Sendai, Miyagi Prefecture, 980-5877 Japan; ^3^ University of Malaya Cancer Research Institute, University of Malaya, Kuala Lumpur, 50603 Malaysia

**Keywords:** tumor microenvironment, cytokines, pancreatic cancer, oxidative stress, metabolic reprogramming

## Abstract

Pancreatic stellate cells (PSC), a prominent stromal cell, contribute to the progression of pancreatic ductal adenocarcinoma (PDAC). We aim to investigate the mechanisms by which PSC promote cell proliferation in PDAC cell lines, BxPC-3 and AsPC-1. PSC-conditioned media (PSC-CM) induced proliferation of these cells in a dose- and time-dependent manner. Nrf2 protein was upregulated and subsequently, its transcriptional activity was increased with greater DNA binding activity and transcription of target genes. Downregulation of Nrf2 led to suppression of PSC-CM activity in BxPC-3, but not in AsPC-1 cells. However, overexpression of Nrf2 alone resulted in increased cell proliferation in both cell lines, and treatment with PSC-CM further enhanced this effect. Activation of Nrf2 pathway resulted in upregulation of metabolic genes involved in pentose phosphate pathway, glutaminolysis and glutathione biosynthesis. Downregulation and inhibition of glucose-6-phosphate-dehydrogenase with siRNA and chemical approaches reduced PSC-mediated cell proliferation. Among the cytokines present in PSC-CM, stromal-derived factor-1 alpha (SDF-1α) and interleukin-6 (IL-6) activated Nrf2 pathway to induce cell proliferation in both cells, as shown with neutralization antibodies, recombinant proteins and signaling inhibitors. Taken together, SDF-1α and IL-6 secreted from PSC induced PDAC cell proliferation via Nrf2-activated metabolic reprogramming and ROS detoxification.

## INTRODUCTION

Pancreatic cancer is the fourth leading cause among men and women in cancer-related deaths, with more than 80% of cases contributed by pancreatic ductal adenocarcinoma (PDAC) [1]. The lethality of PDAC is mainly attributed by its aggressive and invasive growth, rapid development of distant metastases, resistance to therapy, and escape from immune control, all of which have been associated with its dense stroma or desmoplasia [2, 3]. PDAC stroma is very heterogeneous and comprised of pancreatic stellate cells, blood vessels, immune cells, and abundant of extracellular matrix. The cellular composition in PDAC stroma is constantly changing during disease progression [3], hence understanding the molecular mechanism of pancreatic epithelial-stroma interactions can provide useful insights into the biology of this fatal disease.

Pancreatic stellate cells (PSC) are thought to be responsible for the profound desmoplasia observed in PDAC [4]. Under pathological conditions including cancer, quiescent PSC can be activated by reactive oxygen species (ROS) to acquire myofibroblast-like phenotype [5, 6]. Subsequently, activated PSC secrete various soluble factors to modulate PDAC phenotypes including cell proliferation, migration, invasion and metastasis [2, 3, 7–10]. PSC also promote stem cell phenotype in PDAC, which may contribute to the radio- and chemo-resistance frequently observed in patients [11]. Hence, it is crucial to identify and understand the signaling pathways utilized by PSC in its pro-tumorigenic action in PDAC.

Maintaining physiological ROS levels are extremely important to sustain PDAC cellular function [12]. Having constantly higher intracellular ROS levels than normal cells, PDAC requires an effective oxidative defense and cytoprotective system to counteract oxidative stress-mediated cell death. This can be achieved by activation of nuclear factor erythroid 2 (Nrf2) transcription factor, a master regulator of antioxidant-response element (ARE)-driven genes mainly encoding antioxidant and detoxifying enzymes [13]. Nrf2 is abnormally upregulated in PDAC compared to normal pancreatic tissues [14, 15]. In fact, elevated Nrf2 nuclear expression predicts worse survival in PDAC patients [16]. As increased Nrf2 levels are also shown to promote PDAC cell proliferation and acquisition of drug resistance [15], it is unclear if Nrf2 activation can be triggered by extracellular factors, such as PSC.

In this study, we investigated whether PSC secretory factors could activate Nrf2 signaling pathway to induce PDAC cellular proliferation. We demonstrated that secretion from PSC increased Nrf2 nuclear activity and induced transcription of genes involved in ROS detoxification and pentose phosphate pathways. Consequently, intracellular ROS levels were decreased while purine nucleotide synthesis was elevated. Overexpression studies showed that Nrf2 activation was required for PSC-induced PDAC cell proliferation, likely due to the coupling effects of oxidative stress sensing and metabolic reprogramming mechanisms. We further showed that these effects could be induced by the paracrine actions of IL-6 and SDF-1α found in the PSC secretion. Our results suggest a new mechanism by which PSC-derived soluble factors can trigger a redox defense mechanism through activation of Nrf2 signaling, in promoting PDAC aggressiveness.

## RESULTS

### PSC-CM promotes PDAC cell proliferation in a dose- and time-dependent manner

**Figure 1:**
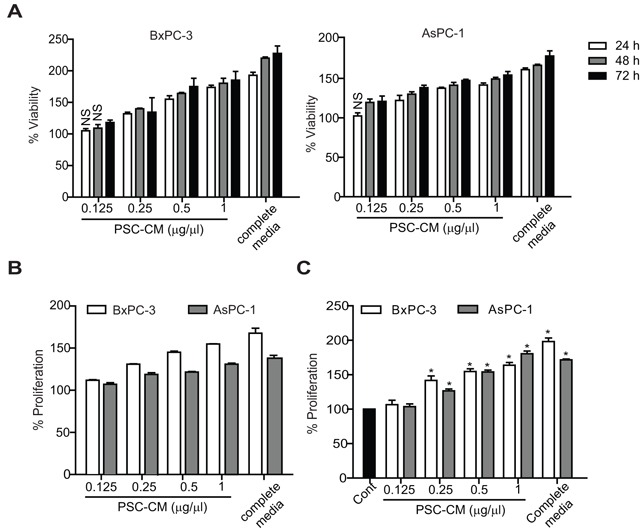
PSC-CM promotes PDAC cell proliferation Cells were treated with PSC-CM for 24-72 h before determination of **A.** cell viability using MTT assay (72 h), **B.** cell proliferation using BrdU assay (72 h), and **C.** trypan-blue cell counting assay (72 h). All experiments were performed in triplicates, and data are expressed as mean ± SD. All data are statistically significant (P<0.05, versus control media (media containing 1% FBS)), unless stated otherwise. NS, not significant.

Treatment of AsPC-1 and BxPC-3 cells with PSC conditioned media (PSC-CM) showed a dose- and time-dependent increase in cell viability (Figure 1A). Notably, 1 μg/μl of PSC-CM at 72 h caused a significant increase in cell viability of BxPC-3 (185 ± 12%) and AsPC-1 (153 ± 4%). The increased cell viability was partly due to the enhanced cell proliferation, as evidenced by increased BrdU incorporation in these cells (BxPC-3: 55 ± 0.1%; AsPC-1: 32 ± 0.1%) (Figure 1B), and increased cell number (BxPC-3: 180 ± 3%; AsPC-1: 157 ± 2%) (Figure 1C), closely within the range induced by complete media. These effects were specific to PSC-CM, as similar results were not observed with conditioned media from fibroblasts derived from normal and cancerous endometrial tissues (Supplementary Figure S1).

### PSC-CM activates intracellular Nrf2 signaling in PDAC cells

**Figure 2:**
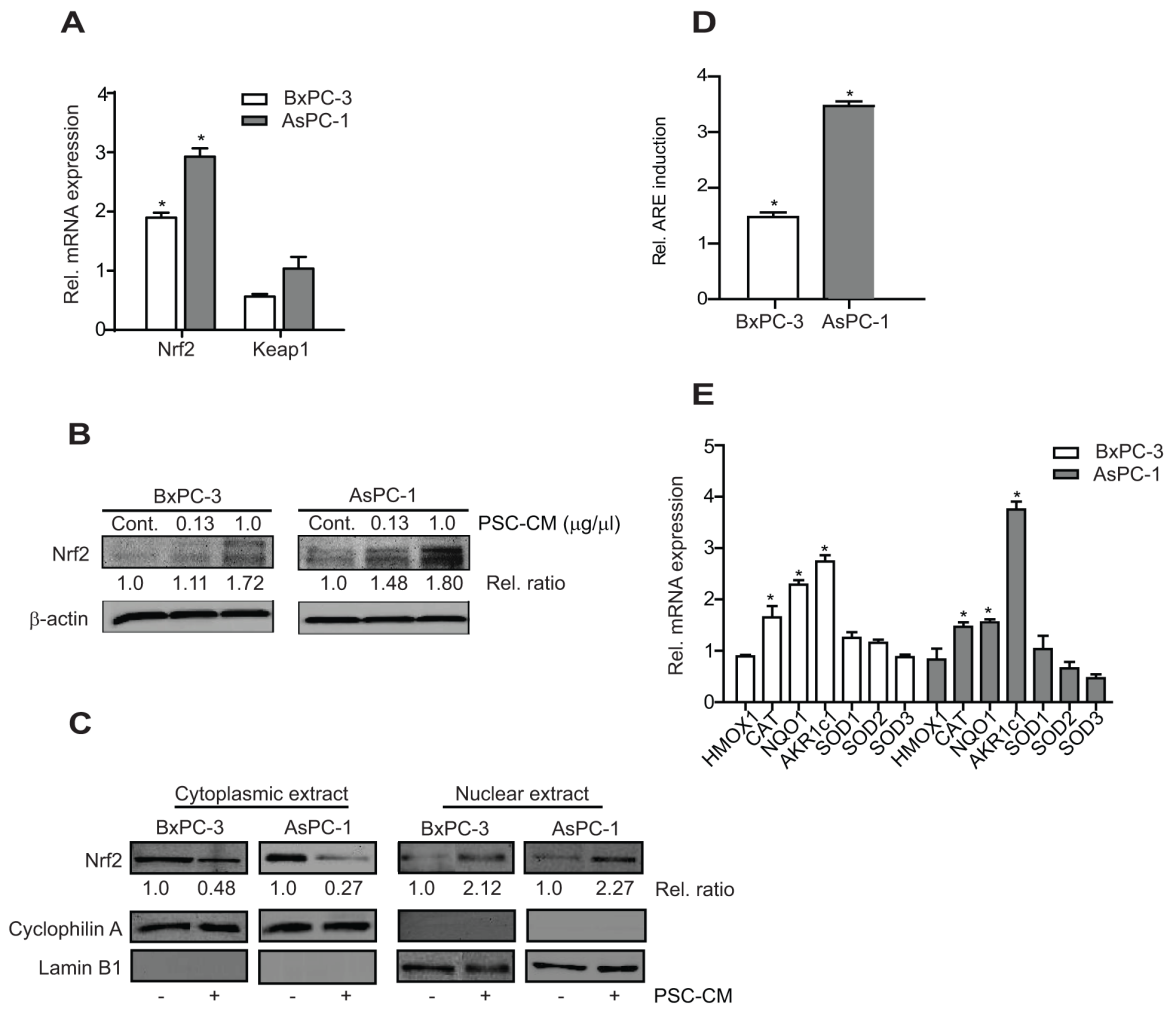
PSC-CM activates Nrf2 signaling in PDAC cells **A, B.** Cells were treated with PSC-CM (1.0 μg/μl, 72 h) before measuring Nrf2 and Keap1 mRNA with qRT-PCR (A), and Nrf2 protein expression with Western blotting (B). Densitometry analysis was shown in relative ratio against control sample. **C.** Nrf2 protein subcellular localization was examined in cells treated with PSC-CM (1.0 μg/μl, 72 h) using immunoblot. Cyclophilin A and lamin B1 were used as loading control for cytoplasmic and nuclear extracts, respectively. Densitometry analysis was shown in relative ratio against untreated cells. **D.** Cells were transfected with ARE promoter reporter construct for 5 h, before treatment with PSC-CM (1.0 μg/μl, 72 h). ARE promoter activity was determined in relative to internal renilla luciferase activity and normalized with protein content between samples. **E.** The mRNA expression of Nrf2-regulated genes in cells after treatment with PSC-CM (1.0 μg/μl, 72 h) was determined using qRT-PCR. All experiments were performed in triplicates, and data are expressed as mean ± SD. *, P<0.05 versus cells treated with media containing 1% FBS.

Treatment of PSC-CM induced higher Nrf2 mRNA expression in both AsPC-1 (2.9-fold) and BxPC-3 cells (1.9-fold) while its negative regulator, Keap1 was downregulated in BxPC-3 cells (0.5-fold) with no significant change in AsPC-1 cells (Figure 2A). Subsequently, Nrf2 protein was upregulated after PSC-CM treatment (Figure 2B), with at least 2-fold increase in Nrf2 localization in the nuclear than in the cytoplasmic extracts of PSC-CM-treated cells (Figure 2C). Using an ARE-promoter luciferase construct, we further showed that PSC-CM induced greater Nrf2 transactivation activity in AsPC-1 cells (3.5-fold) than in BxPC-3 cells (1.5-fold) (Figure 2D). Despite pronounced Nrf2 activation by PSC-CM, only selective Nrf2 cytoprotective genes were induced (Figure 2E). In both cells, AKR1c1 had the greatest induction in BxPC-3 (2.8-fold) and in AsPC-1 cells (4.7-fold). This was followed by NQO1 and CAT expression, both of which were induced at least 2.0-fold in BxPC-3 cells and 1.5-fold in AsPC-1 cells, respectively. Other Nrf2 target genes, HMOX1, SOD1, SOD2 and SOD3 were not significantly affected by PSC-CM treatment. Taken together, these data indicate that extracellular factor like PSC soluble factors could activate Nrf2 transcriptional activity in PDAC cells, and such effects seemed to be more evident in AsPC-1 than in BxPC-3 cells.

### Nrf2 activity is required for PSC-induced PDAC cell proliferation

**Figure 3:**
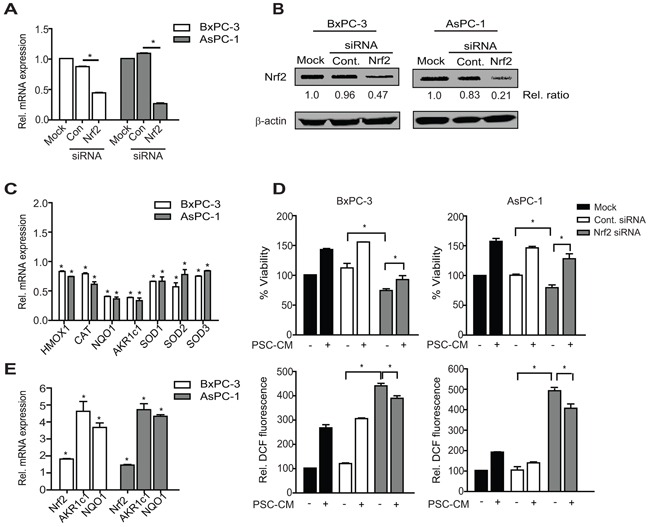
Nrf2 activation is required for PSC-induced PDAC cell proliferation **A, B.** Cells were transfected with control or Nrf2 siRNA (100 nM) for 72 h before determining Nrf2 expression at mRNA (A) and protein expression (B) levels using qRT-PCR and Western blotting, respectively. Densitometry analysis was shown in relative ratio against untreated cells. **C.** The mRNA level of Nrf2 target genes following Nrf2 knockdown were determine. **D, E.** PDAC cells were treated with Nrf2 or control siRNA for 72 h before treatment with PSC-CM (1.0 μg/μl, 72 h). Cell viability (D, top panel) and intracellular ROS levels (D, bottom panel) were measured using MTT and DCF-DA assay, respectively. Nrf2, AKR1c1 and NQO1 mRNA expression was determined using qRT-PCR (E). All experiments were performed in triplicates, and data are expressed as mean ± SD. *, P<0.05.

To determine whether Nrf2 activity is required for PSC-CM-induced cell proliferation, we downregulated Nrf2 protein expression using RNAi approach. Nrf2 mRNA was reduced to 43% and 25% in BxPC-3 and AsPC-1 cells, respectively when compared to untransfected control cells (mock) (P<0.05), with minimal changes in cells transfected with control siRNA (Figure 3A). These results suggest that the effect of Nrf2-siRNA is specific, which subsequently led to 50% and 70% reduction in protein expression in BxPC-3 and AsPC-1 cells, respectively (Figure 3B). Moreover, the transcription of its target genes was also markedly reduced following Nrf2 downregulation, with more than 50% reduction for AKR1c1 and NQO1 (P<0.05 when compared to control siRNA) (Figure 3C).

Reduced Nrf2 protein level led to a greater decrease in cell viability in BxPC-3 (38% inhibition) than in AsPC-1 cells (21% inhibition) (P<0.05 when compared to control siRNA) (Figure 3D), although greater Nrf2 knockdown was observed in the latter. Expectedly, intracellular ROS levels were significantly elevated to almost 5-fold increase in Nrf2-silenced PDAC cells when compared to the control cells (P<0.05) (Figure 3E). In the presence of Nrf2 siRNA, the proliferation-promoting effect of PSC-CM was markedly decreased in BxPC-3 cells (18% increase), when compared to cells treated with control siRNA (41% increase) (P<0.05) (Figure 3D). This was not observed in AsPC-1 cells, where PSC-CM-induced cell proliferation was not significantly affected by Nrf2 downregulation. In both cases, we observed a drop in intracellular ROS levels upon treatment with PSC-CM (Figure 3D), likely due to reactivation of Nrf2 signaling in these cells (Figure 3E). This suggests that Nrf2 activity is required to maintain PDAC cell viability, and to some extent, is required for the increased proliferation effects induced by PSC secretion, at least in BxPC-3 cells.

**Figure 4:**
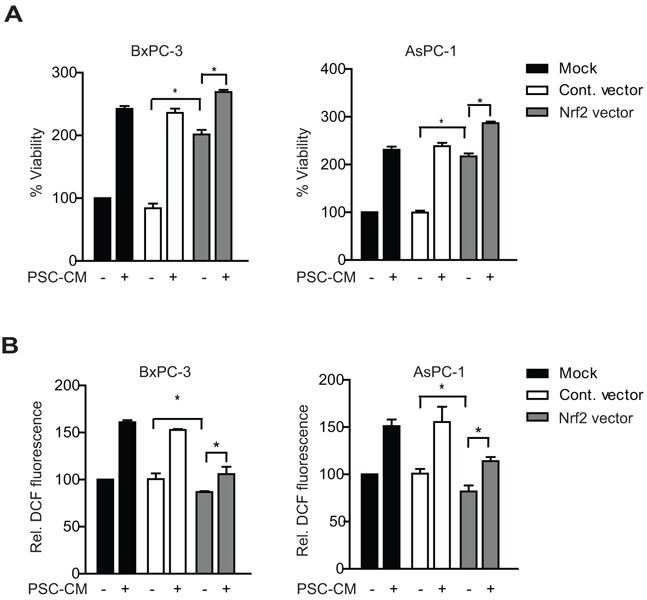
Nrf2 gene overexpression enhances PSC-induced PDAC cell proliferation Cells were transfected with control or Nrf2 expressing vectors for 5 hours, before treatment with PSC-CM (1.0 μg/μl, 72 h). Cell viability **A.** and intracellular ROS levels **B.** were measured using MTT and DCF-DA assay, respectively. All experiments were performed in triplicates and data are expressed as mean ± SD. *, P<0.05.

We further transfected Nrf2-expressing plasmid in both PDAC cells and observed an increase in mRNA levels of Nrf2 (~5 to 6-fold) and its target genes (Supplementary Figure S2A). Nrf2 overexpression resulted in a significant ~2-fold increase in cell viability in both BxPC-3 and AsPC-1 (P<0.05 when compared to control vector) (Figure 4A). Moreover, greater cell proliferation was observed when these cells were treated with PSC-CM, with an additional 33% and 48% increase in BxPC-3 and AsPC-1, respectively (P<0.05 when compared to untreated cells) (Figure 4A). Notably, the proliferation-promoting effects of PSC-CM in Nrf2 overexpressing cells were less evident than in control cells. This could be explained by the moderate induction of Nrf2 mRNA and its target genes by PSC-CM (Supplementary Figure S2B). Despite sustained Nrf2 activation, there was only a slight drop of intracellular ROS levels in Nrf2 overexpressing cells (Figure 4B), indicating that reducing intracellular ROS levels may not be the only mechanism by which activated Nrf2 signaling promote cell proliferation in PDAC cells. Taken together, our data suggests that PSC soluble factors induced PDAC cell proliferation via Nrf2 activation.

### PSC-CM activates metabolic pathways and ROS detoxification via Nrf2

**Figure 5:**
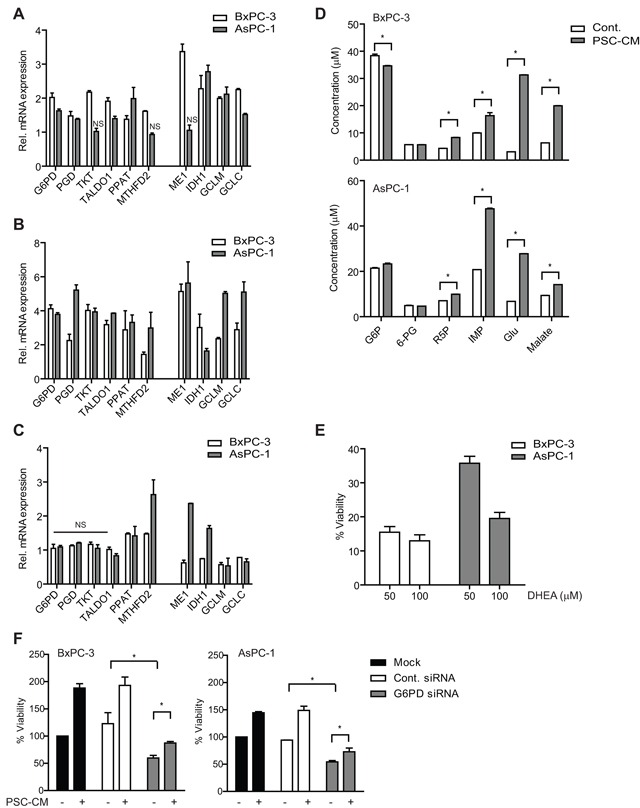
PSC activates metabolic pathways and ROS detoxification in PDAC cells via Nrf2 activity **A–C.** Genes involved in metabolic pathways and ROS detoxification were determined using qPCR in (A) cells treated with control media or PSC-CM (1.0 μg/μl, 72 h), (B) cells transfected with Nrf2 siRNA for 72 h before treated with control media or PSC-CM (1.0 μg/μl, 72 h), and (C) cells transfected with Nrf2 cDNA plasmid for 5 h before treated with control media or PSC-CM (1.0 μg/μl, 72 h). **D.** Extracts from cells treated with either control media or PSC-CM (1.0 μg/μl, 72 h) were subjected to UHPLC-MS/MS analysis to determine the concentration of various metabolites. **E.** Cells were treated with PSC-CM with/without DHEA, before measurement of cell viability. **F.** Following transfection with G6PD or control siRNA, cells were treated with control media or PSC-CM (1.0 μg/μl, 72 h) before measurement of cell viability. Data are expressed as mean ± SD. *, P<0.05 versus cells treated with control media (A-D) or PSC-CM (E). *NS*, not significant.

Nrf2 was recently shown to promote lung cancer cell proliferation by activating metabolic pathways [17]. However, it is unclear if similar mechanisms were adopted by Nrf2 in PDAC cells. We examined key genes involved in three metabolic pathways regulated by Nrf2: pentose phosphate pathway (PPP), glutaminolysis and glutathione biosynthesis pathway. Glucose-6-phosphate dehydrogenase (G6PD), phosphogluconate dehydrogenase (PGD), transketolase (TKT), transaldolase (TALDO1), phosphoribosyl pyrophosphate amidotransferase (PPAT) and methylenetetrahydrofolate dehydrogenase 2 (MTHFD2) are enzymes involved in PPP that generates *de novo* nucleotide and NADPH equivalents. Transcription of these enzymes were significantly induced in both BxPC-3 and AsPC-1 cells following treatment of PSC-CM (P<0.05 when compared to untreated cells), with the exception of TKT and MTHFD2 in AsPC-1 cells (Figure 5A). Genes encoding enzymes for glutaminolysis and glutathione synthesis (malic enzyme 1 (ME1), isocitrate dehydrogenase 1 (IDH1), GCL catalytic subunit (GCLC) and GCL modifier subunit (GCLM)) were also significantly upregulated in these cells, except for ME1 in AsPC-1 cells (Figure 5A). These genes were Nrf2 target genes, as Nrf2 gene knockdown significantly downregulated their expression in both cells (Supplementary Figure S3).

Moreover, we observed a further ~2-fold increase in the expression of these metabolic genes, including TKT, MTHFD2 and ME1 by PSC-CM treatment when Nrf2 was downregulated (Figure 5B). In particular, PGD, MTHFD2, GCLM and GCLC transcription were greater in AsPC-1 than in BxPC-3 cells, which may explain the higher AsPC-1 cell proliferation induced by PSC-CM despite downregulation of Nrf2. Interestingly, when Nrf2 is overexpressed, only PPAT and MTHFD2 were upregulated in both cells, while ME1 and IDH1 were significantly induced in AsPC-1 upon treatment with PSC-CM (Figure 5C). This suggests that PDAC cells may utilize the non-oxidative arm of PPP and glutaminolysis pathways to induce further proliferation when Nrf2 is overexpressed.

Induction of metabolic pathways by PSC-CM led to increased metabolites required in glycolysis, glutaminolysis and nucleotide synthesis (Figure 5D). Ribose 5-phosphate (R5P), a critical substrate for nucleotide synthesis, and inosine 5′-monophosphate (IMP) were significantly increased in both PSC-CM-treated PDAC cells (P<0.05) (Figure 5D). In addition, the concentration of glutamate and malate was increased at least 20-fold and 5-fold, respectively in both cells. To further demonstrate the role of PPP in PSC-induced PDAC cell proliferation, we treated the cells with a G6PD inhibitor (DHEA) in the presence of PSC-CM. BxPC-3 showed significant cell inhibition (~95% inhibition) while AsPC-1 was slightly resistant to the inhibitor (~80% inhibition) at 100 μM (Figure 5E). A more specific downregulation of G6PD protein expression using siRNA led to a significant decrease in cell viability of BxPC-3 (41% inhibition) and AsPC-1 (46% inhibition). Further treatment with PSC-CM only marginally increased the proliferation (22-24% increase) of cells with G6PD knockdown when compared to mock (Figure 5F and Supplementary Figure S4). These data strongly suggest that modulation of metabolic pathways by Nrf2 signaling is critical for PSC-induced cell proliferation in PDAC.

### PSC secrete IL-6 and SDF-1α to activate Nrf2 signaling in PDAC

**Figure 6:**
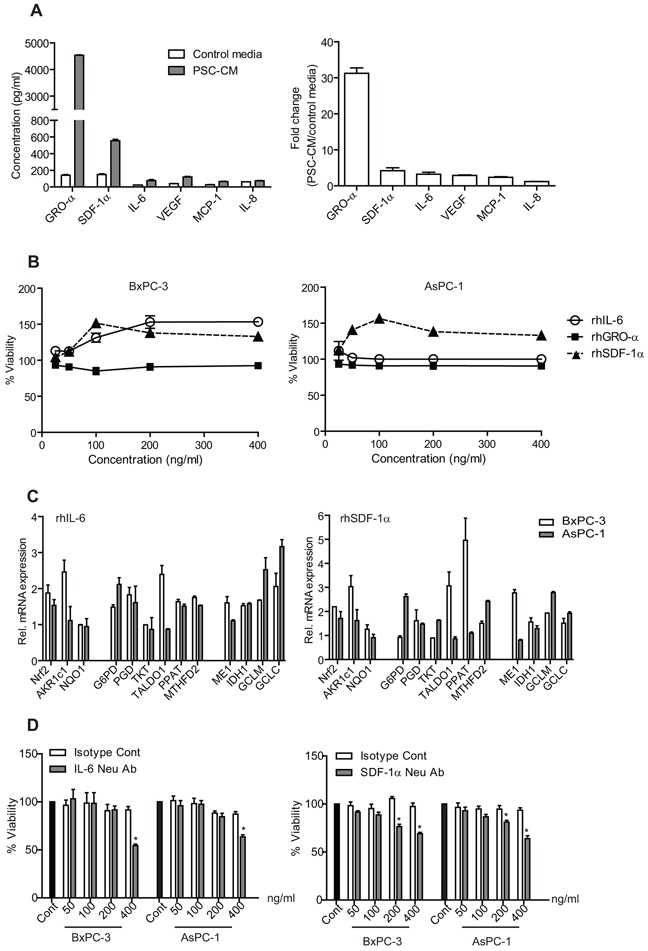
Identification and concentration measurement of soluble factors secreted by PSC **A.** Soluble factors present in PSC-CM and control media were determined using ELISA kits, and expressed in absolute concentration (left) and in fold-change (PSC-CM versus control media) (right). **B.** Cells were treated with recombinant human proteins (rhIL-6, rhGRO-α and rhSDF-1α) for 72 h, before analyzed for their viability. **C.** Selected Nrf2 target mRNA expression were determined using qPCR in cells after treated with rhSDF-1α and rhIL-6. **D.** Cells were treated with either IL-6 or SDF-1 alpha antibodies in the presence of PSC-CM (1.0 μg/μl, 72 h) before determination of cell viability. Data are expressed as mean ± SD. *, P<0.05 versus cells treated with isotype controls.

To determine the possible soluble factors in PSC-CM that may be responsible for activating Nrf2 signaling, we examined a panel of cytokines and growth factors using ELISA kits (Figure 6). Among the soluble factors tested, growth-promoting oncogene alpha (GRO-α) showed the highest level (4534.89 ± 19 pg/ml), followed by stromal-derived factor-1 alpha (SDF-1α) (553.87 ± 17.68 pg/ml) and vascular endothelial growth factor (VEGF) (120.63 ± 4.94 pg/ml) (Figure 6A). When compared to control media, GRO-α remained the cytokine with the greatest fold change (32.3-fold), followed by SDF-1α (4.8-fold) and IL-6 (2.9-fold) (Figure 6A). However, treatment with recombinant protein (rh) GRO-α did not increase PDAC cell proliferation (Figure 6B). Both BxPC-3 and AsPC-1 cells responded to 100 ng/ml rhSDF-1α with 151.5 ± 3.9% and 156.3 ± 2.6%, respectively (Figure 6B), with activation of Nrf2 and its metabolic target genes expression (Figure 6C). Inhibition of SDF-1α effect using neutralizing antibody led to a significant decrease in proliferation in BxPC-3 (46 ± 1.5%) and AsPC-1 (35 ± 2.8%) (Figure 6D).

Interestingly, rhIL-6 treatment induced cell proliferation only in BxPC-3 (153.5 ± 7.8% at 200 ng/ml), but not in AsPC-1 (100.14 ± 3.6% at 200 ng/ml) (Figure 6B). Yet, some Nrf-2 target genes were induced by the treatment (Figure 6C), and neutralization of IL-6 in PSC-CM resulted in a significant decrease in both cells (BxPC-3: 30 ± 1.2%; AsPC-1: 36 ± 2.7%) (Figure 6D). To further investigate whether IL-6 can induce PDAC cell proliferation, we inhibited its downstream signaling using AD412 (a JAK3 inhibitor) and Stattic (a Stat3 inhibitor), and observed a dose-dependent inhibition in both AsPC-1 and BxPC-3 cell proliferation (Supplementary Figure S5). These data suggest that IL-6 and SDF-1α could independently activate Nrf2 signaling and may be partly responsible for PSC-induced cell proliferation.

## DISCUSSION

**Figure 7:**
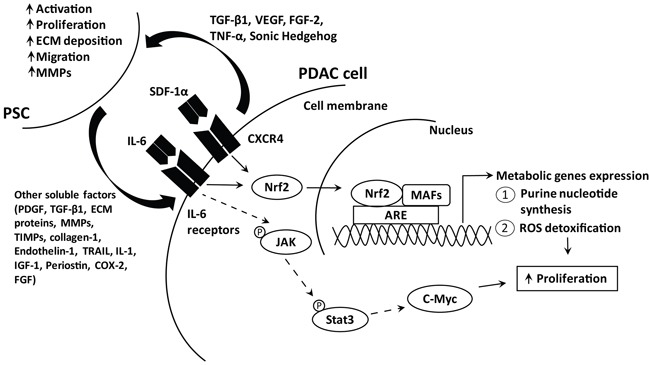
Schematic diagram illustrating the pathways activated by PSC soluble factors to induce PDAC cell proliferation PDAC cells secrete various soluble factors, among which include TGF-β1, VEGF, FGF-2 and sonic hedgehog to recruit and activate PSC. In turn, activated PSC secrete cytokines, matrix metalloproteinases, structural proteins and others, which promote PDAC progression including enhancing proliferation [55]. We showed that IL-6 and SDF-1α secreted by PSC activate their receptor and the subsequent downstream signaling cascade, via Nrf2 transcription factor. Increased Nrf2 activates metabolic gene expression that governs purine nucleotide and NADPH synthesis to promote ROS detoxification and cell proliferation. IL-6 is also known to activate JAK/Stat3 signaling pathway, which induce c-Myc transcription which is important for PDAC cell proliferation.

Our study showed that PSC secretion induced PDAC cellular proliferation by activating antioxidant and metabolic programs via Nrf2. Specifically, IL-6 and SDF-1α promoted Nrf2 transcriptional activity to induce genes involved in ROS detoxification and purine nucleotide synthesis, to create a more reduced intracellular environment that favors cell proliferation (Figure 7). Our study provides further evidence delineating the role of PSC, as an extracellular factor, in activating PDAC intracellular redox signaling to induce cell proliferation.

Desmoplasia in PDAC is mainly due to PSC activation as a result of acute and chronic inflammation within the pancreas [18]. PSC lose their periacinar star-shaped morphology and demonstrate increased proliferation with enhanced production of cytokines, growth factors, miRNAs and deposition of extracellular matrix proteins [18, 19]. Such secretion stimulates PDAC cell proliferation, pancreatosphere formation, invasion and metastasis [7, 20, 21]. PSC co-migrate with PDAC cells to establish a metastatic niche for the tumor cells [22], and resulted in increased metastatic lesion in animal models [3]. PSC may also contribute to hypovascularity and hypoxia, two prominent features in PDAC [23, 24]. Hence, PSC may have an active role in inducing oxidative stress pathways to support a growth-permissive microenvironment for tumor cells.

Despite studies supporting the role of high ROS in PDAC survival [23, 25–27], a recent study suggested that low intracellular ROS levels mediated by Nrf2 activity are essential for PDAC carcinogenesis [28]. Human PDAC tumors and cell lines often exhibit Nrf2 activation and low ROS levels compared to normal pancreatic duct cells [15]. Elevated Nrf2 activity was also observed in other cancers, which was linked to cellular proliferation and development of drug resistance [29–31]. While somatic mutations in Nrf2 and Keap1 genes are rare in PDAC [15], a dysregulated Nrf2/Keap1 system may allow persistent Nrf2 activation, and thus represent an important mechanism by which PDAC sense and adapt to oxidative stress, as observed in other tissues exposed to chronic inflammation [32]. Our data suggests that external factor such as PSC could lead to Nrf2 activation to promote PDAC progression.

Our study showed that PSC soluble factors induced ROS detoxification and purine nucleotide synthesis via Nrf2 activity. As a master regulator of cellular redox homeostasis, Nrf2 upregulates ARE-bearing genes products to confer defense mechanism [33] and induces cellular proliferation via metabolic genes involved in PPP and glutaminolysis [17]. Hence, it is likely that increased synthesis of purine nucleotide, a major product of PPP activation, led to increased PDAC cell proliferation. Moreover, NADPH generated from these pathways is critical for proper cellular redox homeostasis [34]. Complete G6PD knockout in embryonic stem cells led to deprivation of NADPH production, and this resulted to a higher cell kill by potent oxidant [35]. Therefore, PSC-induced PDAC cell proliferation may partly be explained by ROS detoxification and purine nucleotide synthesis.

In the absence of PSC, higher nuclear Nrf2 was observed in AsPC-1 cells than in BxPC-3 cells. Transient silencing of Nrf2 mRNA translation in AsPC-1 cells did not markedly affect proliferation. Yet, Nrf2 overexpression enhanced cell proliferation significantly, indicating that Nrf2 downstream signaling may be consistently activated even in the absence of PSC. The sustained Nrf2 signaling in AsPC-1 may be due to activating KRAS mutation [28]. Oncogenic KRAS is a key driver mutation in PDAC tumorigenesis [36] and is shown to render lower intracellular ROS levels by activating Nrf2 programs [37]. KRAS mutation is also essential in promoting glutaminolysis in PDAC to fuel anabolic processes in supporting cell growth [38, 39]. PDAC relies on non-canonical pathway of glutamine utilization to fuel tricarboxylic acid cycle, in which malate and pyruvate are required to maintain cellular redox state via increasing NADPH/NADP^+^ ratio [40].

Our study suggests that IL-6 and SDF-1α from PSC secretion may be responsible for Nrf2 activation in PDAC cells. The role of IL-6 was rather limited in AsPC-1, probably due to low levels IL-6 receptor expression [41, 42]. Furthermore, IL-6 can partially synergize oncogenic KRAS to activate ROS detoxification program [43]. Nevertheless, inhibition of IL-6 signaling resulted in delayed PDAC progression from pancreatic intraepithelial neoplasia, and reduced primary tumor growth and recurrences *in vivo* [43, 44]. While it is unclear whether Nrf2 is a target gene of IL-6, Nrf2 nuclear translocation and transcriptional activity were significantly reduced in IL-6 knockout mice [45]. Activation of Nrf2 in PDAC may exert a positive feedback to further enhance IL-6 signaling, as there is a known ARE sequence within the promoter of IL-6 [46].

We observed that SDF-1α/CXCR4 signaling activation induced PDAC cell proliferation, regardless of KRAS mutation status. SDF-1α has been implicated in proliferation, invasion, metastasis and chemoresistance of PDAC [47–49]. Nrf2 can directly bind to the promoter of the receptor, CXCR4, and activate its transcription [50]. Moreover, SDF-1α and IL-6 pathways crosstalk leads to the growth promoting effects [51]. More studies are needed to understand how Nrf2 transcriptional activity can be induced by various cytokine signaling, as PSC from different patients may have distinct secretory content with varying growth-promoting effects [2]. Ablation of alpha smooth muscle actin-positive myofibroblasts in a transgenic PDAC mouse model was shown to exacerbate tumor progression leading to decreased overall survival, arguing the exact role of PSC in PDAC progression [52].

In conclusion, our study provides the evidence that PSC-derived soluble factors are contributing to the maintenance of a reduced intracellular environment and metabolic reprogramming, which is favorable for PDAC development and progression. Delineating the molecular convergence downstream of Nrf2 pathway may provide further insights into novel therapeutic targets, in the effort to improve prognosis of PDAC.

## MATERIALS AND METHODS

### Chemicals

Dehydroisoandrosterone (DHEA) was purchased from Acros Organics (Geel, Belgium) and 2′,7′-dichlorofluorescein diacetate (DCF-DA) was obtained from Life Technologies (CA, USA). Glucose 6-phosphate, 6-phosphogluconate, ribose 5-phosphate, inosine 5′-monophosphate, glutamate, malate, 4-nitrobenzoic acid and 3-(4,5-dimethylthiazol-2-yl)-2 5-diphenyltetrazolium bromide (MTT) were purchased from Sigma-Aldrich (MO, USA). Stattic and AD412 were obtained from Santa Cruz Biotechnology (Texas, USA).

### Cell culture

Human pancreatic adenocarcinoma cells (PDAC) such as BxPC-3 and AsPC-1, and immortalized human normal endometrial fibroblast cells (T-HESC) were purchased from American Type Culture Collection (VA, USA). Human pancreatic stellate cells (PSC) were isolated from the resected pancreas tissues of patients undergoing operation for pancreatic cancer as previously described [53], under the approval by the Ethics Committee of Tohoku University School of Medicine (article#: 2009-79 and 2014-1-132), while human endometrial adenocarcinoma primary fibroblast cells (EC6/Fib) were isolated from resected endometrium tissues [54]. PDAC and EC6/Fib were maintained in RPMI-1640, while PSC were maintained in DMEM/F-12 media, all supplemented with 10% fetal bovine serum and 1% penicillin-streptomycin (Life Technologies) at 37°C in 95% humidified atmosphere of 5% CO_2_.

### Preparation of fibroblasts conditioned media

Fibroblasts (PSC, T-HESC and EC6/Fib) were grown with complete media to 70-80% confluency in 48 h before changing to media with 0% or 1% FBS for additional 72 h. Supernatant were concentrated with Amicon Ultra-15 centrifugal filter (Merck Millipore, MA, USA) by centrifugation at 5000 × g at 4°C for 1 h, and the protein was quantified using Bradford assay (BioRad, CA, USA).

### Cell viability and proliferation assays

Briefly, 1 × 10^4^ cells were plated into 96-well plate for 24 h and serum-starved for additional 24 h, before treated with complete media, control media (media containing 1% FBS) or fibroblast conditioned media (1.0 μg/μl) with or without chemicals, recombinant proteins or neutralizing antibodies (BioLegend, CA, USA) for subsequent 24-72 h. At the end of treatment, MTT solution (5 mg/ml) was added into each well, followed by 4 h incubation at 37°C. The formazan crystals were dissolved with sodium dodecyl sulfate before reading the absorbance using CHAMELEON™V plate reader (Hidex, Finland) at 570 nm with reference wavelength of 630 nm.

To measure cell proliferation, we determined BrdU incorporation into the cellular DNA using an ELISA-based approach (Cell Signaling Technology, MA, USA). Cells were seeded and treated as described above, and at the end of treatment, BrdU solution was added for 24 h. Following fixation and denaturation, the cells were labeled with antibodies and washed before added with TMB substrate. Color development was terminated with a STOP solution, and measured at 450 nm using CHAMELEON™V plate reader. In addition, the cells were also stained with 0.04% trypan blue to count for number of viable cells, using neubauer improved hemocytometer (Sigma Aldrich).

### Intracellular ROS measurement

The cells were treated with 10 μM DCF-DA for 20 min at 37°C in a dark, before lysed with 1% Triton-X 100 for 5 min at room temperature. The fluorescence was observed with inverted fluorescence microscope (Nikon Eclipse T*i*, NY, USA) and measured at 485/535 nm using CHAMELEON™V plate reader.

### Real time quantitative RT-PCR (qRT-PCR)

Total RNA was extracted using TRIzol (Life Technologies) and 1 μg RNA was converted into cDNA using RevertAid first strand cDNA synthesis kit (Thermo Scientific, MA, USA). The forward and reverse primers sequence used are listed in Supplementary Table S1. qRT-PCR was performed using an ABI StepOne Plus (Applied Biosystem, CA, USA) in 40 cycles using 5x Evagreen qPCR mix (Solis BioDyne, Tartu, Estonia), 10 pmol/μl forward and reverse primers, 10 ng/μl cDNA and water. Each data point was performed in triplicates and normalized with housekeeping gene, GAPDH. Relative expression of treated cells was calculated against untreated cells using the ΔΔC(t) method.

### Western blot analysis

Total cell lysates were harvested using lysis buffer containing 0.1% Triton-X 100, 0.1% SDS, 50 mM Tris, 150 mM NaCl and protease and phosphatase inhibitors, while the cytoplasmic and nuclear cell lysates were prepared using NE-PER kit (Thermo Scientific). Protein concentration was quantified using Bradford assay (BioRad). Proteins (25 μg) were resolved on 10-12% SDS-PAGE before transferred to polyvinylidene difluoride membrane. The membrane was first blocked in 5% non-fat dry milk before probed with antibodies of human Nrf2 (Novus Biologicals, CO, USA), lamin B1, cyclophilin A (Cell Signaling Technology) and β-actin (Santa Cruz Biotechnology, Texas, USA). After washing, the blots were probed with secondary antibody conjugated with horseradish peroxidase (Santa Cruz Biotechnology). Detection was performed using ECL detection reagent (Amersham, Sweden); visualization and densitometry analysis was performed using gel documentation system (UVP, CA, USA).

### ARE-promoter transactivation activity

Briefly, 1 × 10^4^ cells were co-transfected with pGL4.37[*luc2P*/ARE/Hygro] containing a firefly luciferase construct, and pGL4.74[hRluc/TK] (Promega, Madison, USA) containing a renilla luciferase construct, using Lipofectamine LTX (Life Technologies). After 5 h of transfection, the cells were treated with control media or PSC-CM (1.0 μg/μl) for 72 h. Luciferase activities were measured using Tecan multiplate reader (Männedorf, Switzerland) and relative ARE induction was determined by calculating the ratio of firefly luciferase reading with renilla luciferase reading, after normalized with protein content.

### Transient Nrf2 and G6PD gene silencing and overexpression

Approximately 1 × 10^4^ cells were seeded in 96-well plate in complete media without antibiotic overnight. Before transfection, 100 nM of ON-TARGETplus siRNA targeting Nrf2 or G6PD, ON-TARGETplus siControl and DharmaFECT 2 (Thermo Scientific) were diluted individually in OptiMEM (Life Technologies) for 5 min. Both siRNA and siControl solution were mixed with DharmaFECT 2 solution for additional 20 min before treating the cells in the presence of complete media without antibiotics for 48 h. To induce Nrf2 gene expression, 1 × 10^4^ cells were seeded in 96-well plate in complete media. Before transfection, 100 ng of Nrf2 ORF and control vector (OriGene, MD, USA), and Lipofectamine LTX with PLUS reagent (Life Technologies) were individually incubated with OptiMEM (Life Technologies) for 30 min. Both vectors were mixed with Lipofectamine solution before transfection for 5 h.

### Metabolite extraction and measurement

The metabolite extraction was prepared according to Sheikh *et al* [55]. Briefly, 1 × 10^7^ cells were resuspended in 150 μl water and lysed with two cycles of freeze-thaw, followed by ultrasonic homogenization for 30 sec on ice. Cell lysates were added with methanol containing internal standard (4-nitrobenzoic acid), vortexed and incubated on ice for 15 min. Subsequently, chloroform was added into the solution, prior to vortex and centrifugation at 13000 rpm for 10 min at 15°C. The two phases separated were transferred to a fresh tube, before mixing with chilled acetonitrile and further incubation at −80°C for 2 h. Following centrifugation at 13000 rpm for 10 min at 4°C, the supernatants were transferred to fresh tubes, dried under vacuum and resuspended in 50% acetonitrile/water. Samples were injected into Thermo Scientific Dionex Ultimate 3000 Rapid Separation LC coupled to an Orbitrap Fusion™ Tribrid™ mass spectrometer (Thermo Scientific) and separation was achieved on a Hypersil GOLD aqueous column (Thermo Scientific) using a 20 min gradient with mobile phases consisting of 95% water and 5% acetonitrile. MS analysis was performed using the Thermo Scientific Xcalibur 3.0 software.

### Statistical analysis

Statistical analysis was performed using GraphPad Prism version 5 (GraphPad, CA, USA). The difference between two groups was analyzed by two-tailed Student t test. The difference between three or more groups was analyzed by one-way analysis of variance multiple comparisons (ANOVA). Bars in the figures were considered statistically significant different at P<0.05. Data shown as mean ± standard deviation (SD).

## SUPPLEMENTARY FIGURES AND TABLE



## References

[1] Siegel RL, Miller KD, Jemal A (2015). Cancer statistics, 2015. CA Cancer J Clin.

[2] Hwang RF, Moore T, Arumugam T, Ramachandran V, Amos KD, Rivera A, Ji B, Evans DB, Logsdon CD (2008). Cancer-associated stromal fibroblasts promote pancreatic tumor progression. Cancer Res.

[3] Vonlaufen A, Joshi S, Qu C, Phillips PA, Xu Z, Parker NR, Toi CS, Pirola RC, Wilson JS, Goldstein D, Apte MV (2008). Pancreatic stellate cells: partners in crime with pancreatic cancer cells. Cancer Res.

[4] Kikuta K, Masamune A, Watanabe T, Ariga H, Itoh H, Hamada S, Satoh K, Egawa S, Unno M, Shimosegawa T (2010). Pancreatic stellate cells promote epithelial-mesenchymal transition in pancreatic cancer cells. Biochem Biophys Res Commun.

[5] Kikuta K, Masamune A, Satoh M, Suzuki N, Satoh K, Shimosegawa T (2006). Hydrogen peroxide activates activator protein-1 and mitogen-activated protein kinases in pancreatic stellate cells. Mol Cell Biochem.

[6] Costa A, Scholer-Dahirel A, Mechta-Grigoriou F (2014). The role of reactive oxygen species and metabolism on cancer cells and their microenvironment. Semin Cancer Biol.

[7] Bachem MG, Schunemann M, Ramadani M, Siech M, Beger H, Buck A, Zhou S, Schmid-Kotsas A, Adler G (2005). Pancreatic carcinoma cells induce fibrosis by stimulating proliferation and matrix synthesis of stellate cells. Gastroenterology.

[8] Lu J, Zhou S, Siech M, Habisch H, Seufferlein T, Bachem MG (2014). Pancreatic stellate cells promote hapto-migration of cancer cells through collagen I-mediated signalling pathway. Br J Cancer.

[9] Mantoni TS, Lunardi S, Al-Assar O, Masamune A, Brunner TB (2011). Pancreatic stellate cells radioprotect pancreatic cancer cells through beta1-integrin signaling. Cancer Res.

[10] Masamune A, Watanabe T, Kikuta K, Shimosegawa T (2009). Roles of pancreatic stellate cells in pancreatic inflammation and fibrosis. Clin Gastroenterol Hepatol.

[11] Hamada S, Masamune A, Shimosegawa T (2013). Alteration of pancreatic cancer cell functions by tumor-stromal cell interaction. Front Physiol.

[12] Vaquero EC, Edderkaoui M, Pandol SJ, Gukovsky I, Gukovskaya AS (2004). Reactive oxygen species produced by NAD(P)H oxidase inhibit apoptosis in pancreatic cancer cells. J Biol Chem.

[13] Bryan HK, Olayanju A, Goldring CE, Park BK (2013). The Nrf2 cell defence pathway: Keap1-dependent and -independent mechanisms of regulation. Biochem Pharmacol.

[14] Homma S, Ishii Y, Morishima Y, Yamadori T, Matsuno Y, Haraguchi N, Kikuchi N, Satoh H, Sakamoto T, Hizawa N, Itoh K, Yamamoto M (2009). Nrf2 enhances cell proliferation and resistance to anticancer drugs in human lung cancer. Clin Cancer Res.

[15] Lister A, Nedjadi T, Kitteringham NR, Campbell F, Costello E, Lloyd B, Copple IM, Williams S, Owen A, Neoptolemos JP, Goldring CE, Park BK (2011). Nrf2 is overexpressed in pancreatic cancer: implications for cell proliferation and therapy. Mol Cancer.

[16] Soini Y, Eskelinen M, Juvonen P, Karja V, Haapasaari KM, Saarela A, Karihtala P (2014). Nuclear Nrf2 expression is related to a poor survival in pancreatic adenocarcinoma. Pathol Res Pract.

[17] Mitsuishi Y, Taguchi K, Kawatani Y, Shibata T, Nukiwa T, Aburatani H, Yamamoto M, Motohashi H (2012). Nrf2 redirects glucose and glutamine into anabolic pathways in metabolic reprogramming. Cancer Cell.

[18] Feig C, Gopinathan A, Neesse A, Chan DS, Cook N, Tuveson DA (2012). The pancreas cancer microenvironment. Clin Cancer Res.

[19] Masamune A, Kikuta K, Watanabe T, Satoh K, Hirota M, Hamada S, Shimosegawa T (2009). Fibrinogen induces cytokine and collagen production in pancreatic stellate cells. Gut.

[20] Erkan M, Adler G, Apte MV, Bachem MG, Buchholz M, Detlefsen S, Esposito I, Friess H, Gress TM, Habisch HJ, Hwang RF, Jaster R, Kleeff J, Kloppel G, Kordes C, Logsdon CD (2012). StellaTUM: current consensus and discussion on pancreatic stellate cell research. Gut.

[21] Ali S, Suresh R, Banerjee S, Bao B, Xu Z, Wilson J, Philip PA, Apte M, Sarkar FH (2015). Contribution of microRNAs in understanding the pancreatic tumor microenvironment involving cancer associated stellate and fibroblast cells. American journal of cancer research.

[22] Xu Z, Vonlaufen A, Phillips PA, Fiala-Beer E, Zhang X, Yang L, Biankin AV, Goldstein D, Pirola RC, Wilson JS, Apte MV (2010). Role of pancreatic stellate cells in pancreatic cancer metastasis. Am J Pathol.

[23] Olive KP, Jacobetz MA, Davidson CJ, Gopinathan A, McIntyre D, Honess D, Madhu B, Goldgraben MA, Caldwell ME, Allard D, Frese KK, Denicola G, Feig C, Combs C, Winter SP, Ireland-Zecchini H (2009). Inhibition of Hedgehog signaling enhances delivery of chemotherapy in a mouse model of pancreatic cancer. Science.

[24] Koong AC, Mehta VK, Le QT, Fisher GA, Terris DJ, Brown JM, Bastidas AJ, Vierra M (2000). Pancreatic tumors show high levels of hypoxia. Int J Radiat Oncol Biol Phys.

[25] Cullen JJ, Weydert C, Hinkhouse MM, Ritchie J, Domann FE, Spitz D, Oberley LW (2003). The role of manganese superoxide dismutase in the growth of pancreatic adenocarcinoma. Cancer Res.

[26] Mouria M, Gukovskaya AS, Jung Y, Buechler P, Hines OJ, Reber HA, Pandol SJ (2002). Food-derived polyphenols inhibit pancreatic cancer growth through mitochondrial cytochrome C release and apoptosis. Int J Cancer.

[27] Teoh ML, Sun W, Smith BJ, Oberley LW, Cullen JJ (2007). Modulation of reactive oxygen species in pancreatic cancer. Clin Cancer Res.

[28] DeNicola GM, Karreth FA, Humpton TJ, Gopinathan A, Wei C, Frese K, Mangal D, Yu KH, Yeo CJ, Calhoun ES, Scrimieri F, Winter JM, Hruban RH, Iacobuzio-Donahue C, Kern SE, Blair IA (2011). Oncogene-induced Nrf2 transcription promotes ROS detoxification and tumorigenesis. Nature.

[29] Shibata T, Kokubu A, Gotoh M, Ojima H, Ohta T, Yamamoto M, Hirohashi S (2008). Genetic alteration of Keap1 confers constitutive Nrf2 activation and resistance to chemotherapy in gallbladder cancer. Gastroenterology.

[30] Shibata T, Ohta T, Tong KI, Kokubu A, Odogawa R, Tsuta K, Asamura H, Yamamoto M, Hirohashi S (2008). Cancer related mutations in NRF2 impair its recognition by Keap1-Cul3 E3 ligase and promote malignancy. Proc Natl Acad Sci U S A.

[31] Stacy DR, Ely K, Massion PP, Yarbrough WG, Hallahan DE, Sekhar KR, Freeman ML (2006). Increased expression of nuclear factor E2 p45-related factor 2 (NRF2) in head and neck squamous cell carcinomas. Head Neck.

[32] Stachel I, Geismann C, Aden K, Deisinger F, Rosenstiel P, Schreiber S, Sebens S, Arlt A, Schafer H (2014). Modulation of nuclear factor E2-related factor-2 (Nrf2) activation by the stress response gene immediate early response-3 (IER3) in colonic epithelial cells: a novel mechanism of cellular adaption to inflammatory stress. J Biol Chem.

[33] Murakami S, Motohashi H (2015). Roles of NRF2 in cell proliferation and differentiation. Free Radic Biol Med.

[34] Stanton RC (2012). Glucose-6-phosphate dehydrogenase, NADPH, and cell survival. IUBMB Life.

[35] Pandolfi PP, Sonati F, Rivi R, Mason P, Grosveld F, Luzzatto L (1995). Targeted disruption of the housekeeping gene encoding glucose 6-phosphate dehydrogenase (G6PD): G6PD is dispensable for pentose synthesis but essential for defense against oxidative stress. EMBO J.

[36] Collins MA, Pasca di Magliano M (2013). Kras as a key oncogene and therapeutic target in pancreatic cancer. Front Physiol.

[37] Kong B, Qia C, Erkan M, Kleeff J, Michalski CW (2013). Overview on how oncogenic Kras promotes pancreatic carcinogenesis by inducing low intracellular ROS levels. Front Physiol.

[38] Ying H, Kimmelman AC, Lyssiotis CA, Hua S, Chu GC, Fletcher-Sananikone E, Locasale JW, Son J, Zhang H, Coloff JL, Yan H, Wang W, Chen S, Viale A, Zheng H, Paik JH (2012). Oncogenic Kras maintains pancreatic tumors through regulation of anabolic glucose metabolism. Cell.

[39] Wise DR, Thompson CB (2010). Glutamine addiction: a new therapeutic target in cancer. Trends Biochem Sci.

[40] Son J, Lyssiotis CA, Ying H, Wang X, Hua S, Ligorio M, Perera RM, Ferrone CR, Mullarky E, Shyh-Chang N, Kang Y, Fleming JB, Bardeesy N, Asara JM, Haigis MC, DePinho RA (2013). Glutamine supports pancreatic cancer growth through a KRAS-regulated metabolic pathway. Nature.

[41] Masui T, Hosotani R, Doi R, Miyamoto Y, Tsuji S, Nakajima S, Kobayashi H, Koizumi M, Toyoda E, Tulachan SS, Imamura M (2002). Expression of IL-6 receptor in pancreatic cancer: involvement in VEGF induction. Anticancer Res.

[42] Miyamoto Y, Hosotani R, Doi R, Wada M, Ida J, Tsuji S, Kawaguchi M, Nakajima S, Kobayashi H, Masui T, Imamura M (2001). Interleukin-6 inhibits radiation induced apoptosis in pancreatic cancer cells. Anticancer Res.

[43] Zhang Y, Yan W, Collins MA, Bednar F, Rakshit S, Zetter BR, Stanger BZ, Chung I, Rhim AD, di Magliano MP (2013). Interleukin-6 is required for pancreatic cancer progression by promoting MAPK signaling activation and oxidative stress resistance. Cancer Res.

[44] Goumas FA, Holmer R, Egberts JH, Gontarewicz A, Heneweer C, Geisen U, Hauser C, Mende MM, Legler K, Rocken C, Becker T, Waetzig GH, Rose-John S, Kalthoff H (2015). Inhibition of IL-6 signaling significantly reduces primary tumor growth and recurrencies in orthotopic xenograft models of pancreatic cancer. Int J Cancer.

[45] Tran HY, Shin EJ, Saito K, Nguyen XK, Chung YH, Jeong JH, Bach JH, Park DH, Yamada K, Nabeshima T, Yoneda Y, Kim HC (2011). Protective potential of IL-6 against trimethyltin-induced neurotoxicity in vivo. Free Radic Biol Med.

[46] Wruck CJ, Streetz K, Pavic G, Gotz ME, Tohidnezhad M, Brandenburg LO, Varoga D, Eickelberg O, Herdegen T, Trautwein C, Cha K, Kan YW, Pufe T (2011). Nrf2 induces interleukin-6 (IL-6) expression via an antioxidant response element within the IL-6 promoter. J Biol Chem.

[47] Gao Z, Wang X, Wu K, Zhao Y, Hu G (2010). Pancreatic stellate cells increase the invasion of human pancreatic cancer cells through the stromal cell-derived factor-1/CXCR4 axis. Pancreatology.

[48] Matsuo Y, Ochi N, Sawai H, Yasuda A, Takahashi H, Funahashi H, Takeyama H, Tong Z, Guha S (2009). CXCL8/IL-8 and CXCL12/SDF-1alpha co-operatively promote invasiveness and angiogenesis in pancreatic cancer. Int J Cancer.

[49] Shakir M, Tang D, Zeh HJ, Tang SW, Anderson CJ, Bahary N, Lotze MT (2015). The chemokine receptors CXCR4/CXCR7 and their primary heterodimeric ligands CXCL12 and CXCL12/high mobility group box 1 in pancreatic cancer growth and development: finding flow. Pancreas.

[50] Tsai JJ, Dudakov JA, Takahashi K, Shieh JH, Velardi E, Holland AM, Singer NV, West ML, Smith OM, Young LF, Shono Y, Ghosh A, Hanash AM, Tran HT, Moore MA, van den Brink MR (2013). Nrf2 regulates haematopoietic stem cell function. Nat Cell Biol.

[51] Zhang H, Wu H, Guan J, Wang L, Ren X, Shi X, Liang Z, Liu T (2014). Paracrine SDF-1alpha signaling mediates the effects of PSCs on GEM chemoresistance through an IL-6 autocrine loop in pancreatic cancer cells. Oncotarget.

[52] Ozdemir BC, Pentcheva-Hoang T, Carstens JL, Zheng X, Wu CC, Simpson TR, Laklai H, Sugimoto H, Kahlert C, Novitskiy SV, De Jesus-Acosta A, Sharma P, Heidari P, Mahmood U, Chin L, Moses HL (2014). Depletion of carcinoma-associated fibroblasts and fibrosis induces immunosuppression and accelerates pancreas cancer with reduced survival. Cancer Cell.

[53] Masamune A, Kikuta K, Watanabe T, Satoh K, Hirota M, Shimosegawa T (2008). Hypoxia stimulates pancreatic stellate cells to induce fibrosis and angiogenesis in pancreatic cancer. Am J Physiol Gastrointest Liver Physiol.

[54] Subramaniam KS, Tham ST, Mohamed Z, Woo YL, Mat Adenan NA, Chung I (2013). Cancer-associated fibroblasts promote proliferation of endometrial cancer cells. PloS one.

[55] Sheikh KD, Khanna S, Byers SW, Fornace A, Cheema AK (2011). Small molecule metabolite extraction strategy for improving LC/MS detection of cancer cell metabolome. J Biomol Tech.

