# Supplementary material for: Soluble factors from stellate cells induce pancreatic cancer cell proliferation via Nrf2-activated metabolic reprogramming and ROS detoxification
Source: Oncotarget. 2016 May 4;7(24):36719–32. doi: 10.18632/oncotarget.9165 (PMC5095034; doi:10.18632/oncotarget.9165)
Supplement: Supplementary file 1 [file oncotarget-07-36719-s001.pdf]

SUPPLEMENTARY FIGURES AND TABLE

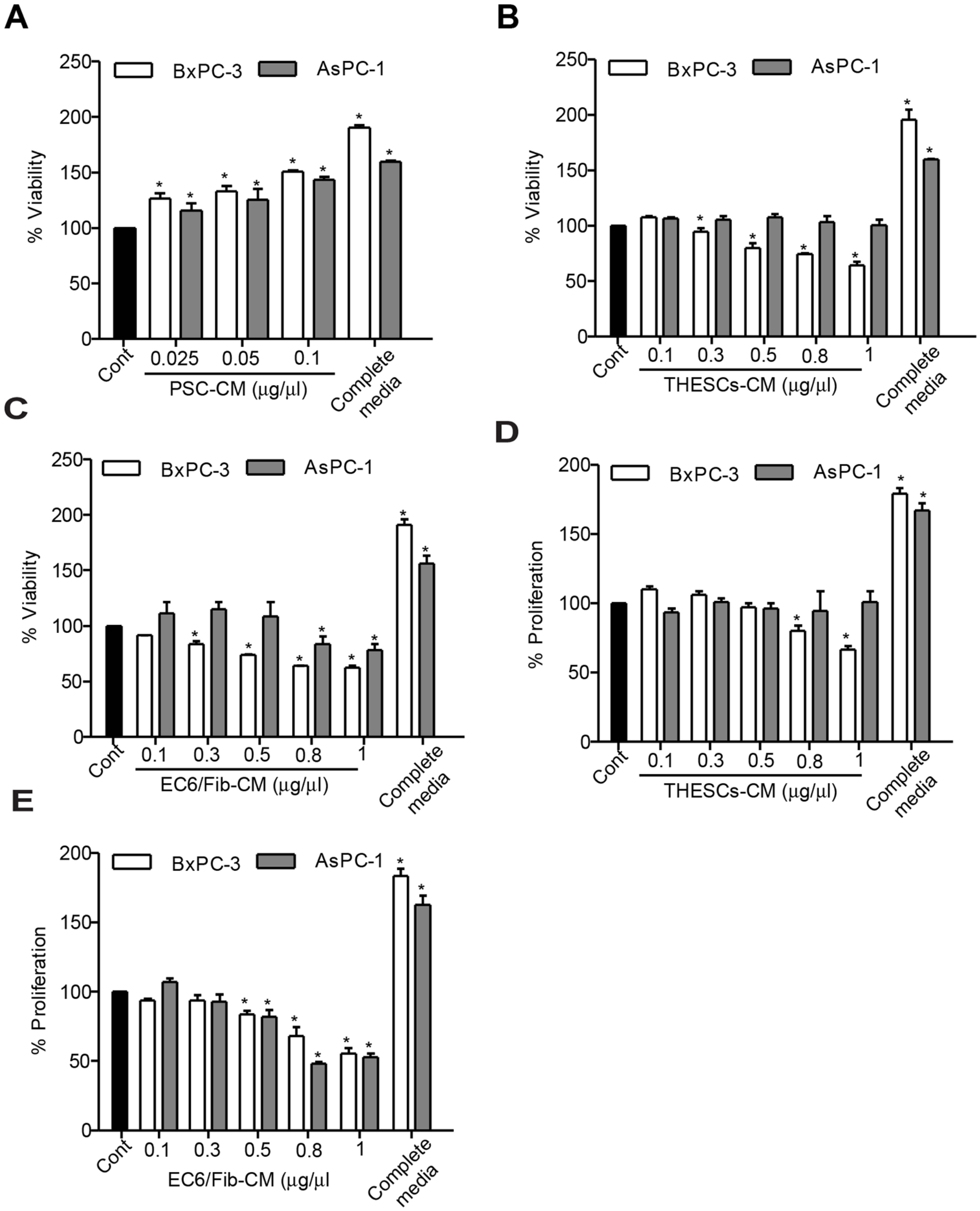

Supplementary Figure S1: The effect of various fibroblasts-derived conditioned media on PDAC cell proliferation. PSC were culture in media devoid of FBS (0% FBS) for 72 hours, while normal endometrial fibroblasts (THESC) and cancer-associated fibroblasts from endometrial cancer (EC6/Fib) were cultured in media containing 1% FBS for 72 hours, before collection of conditioned media. PDAC cells were treated with these different conditioned media for 72 hours before analysis of cell viability using MTT assay **A–C.** cell counting **D–E.** All experiments were performed in triplicates, and data are expressed as mean ± SD. All data are statistically significant (P<0.05 versus cells treated with blank media).

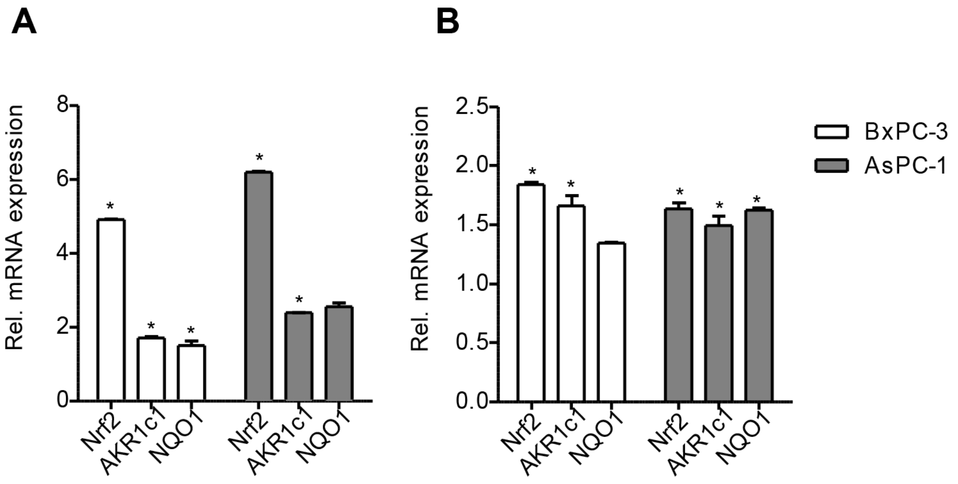

Supplementary Figure S2: Expression of Nrf2 and its target genes in cells overexpressed with Nrf2 cDNA vector. A. Cells were transfected with control or Nrf2 cDNA vectors for 5 h, before subjected to qPCR analysis. **B.** Cells were transfected with Nrf2 cDNA vectors for 5 h, followed by treatment with PSC-CM (1.0 μg/μl, 72 h). The mRNA levels of genes were measured with qPCR. Data are expressed as mean ± SD.*, P<0.05.

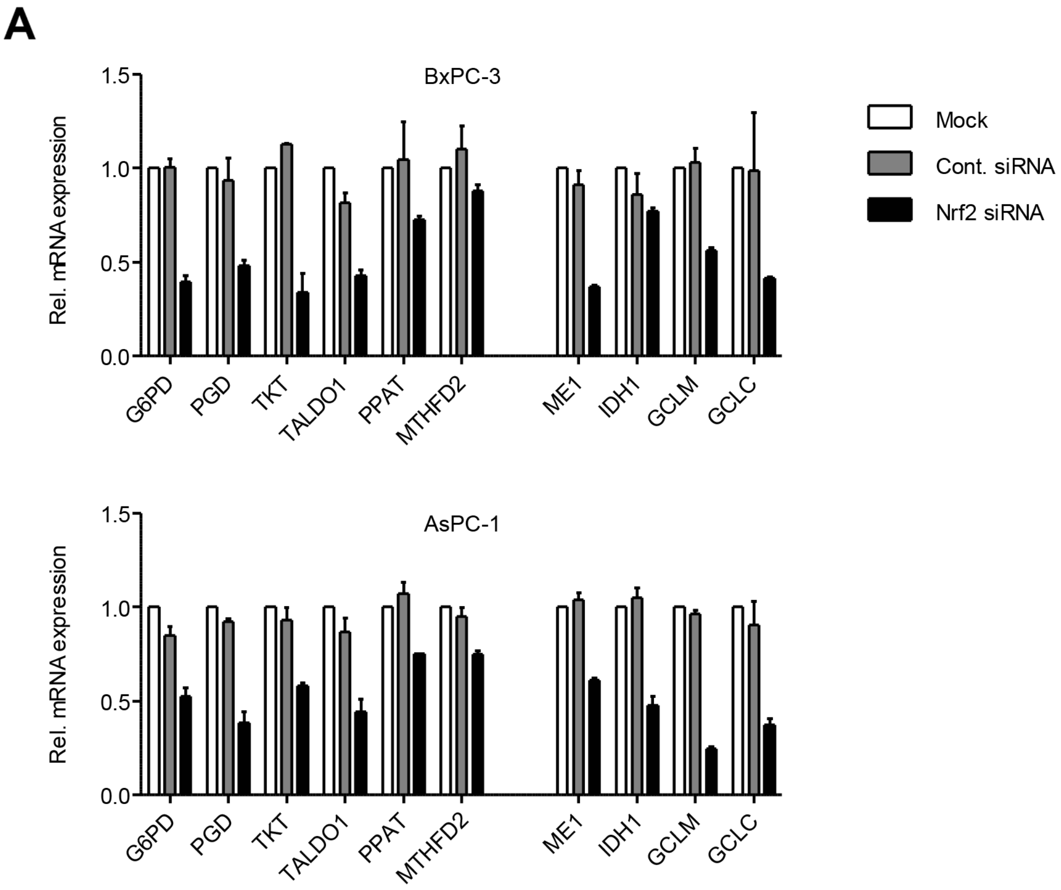

Supplementary Figure S3: Nrf2 induces metabolic genes expression. A. After Nrf2 gene was silenced, the mRNA level of Nrf2-mediated metabolic genes was quantified using qRT-PCR. All experiments were performed in triplicates, and representative data are shown. Data are expressed as mean ± SD. *, P < 0.05 versus control siRNA transfected cells.

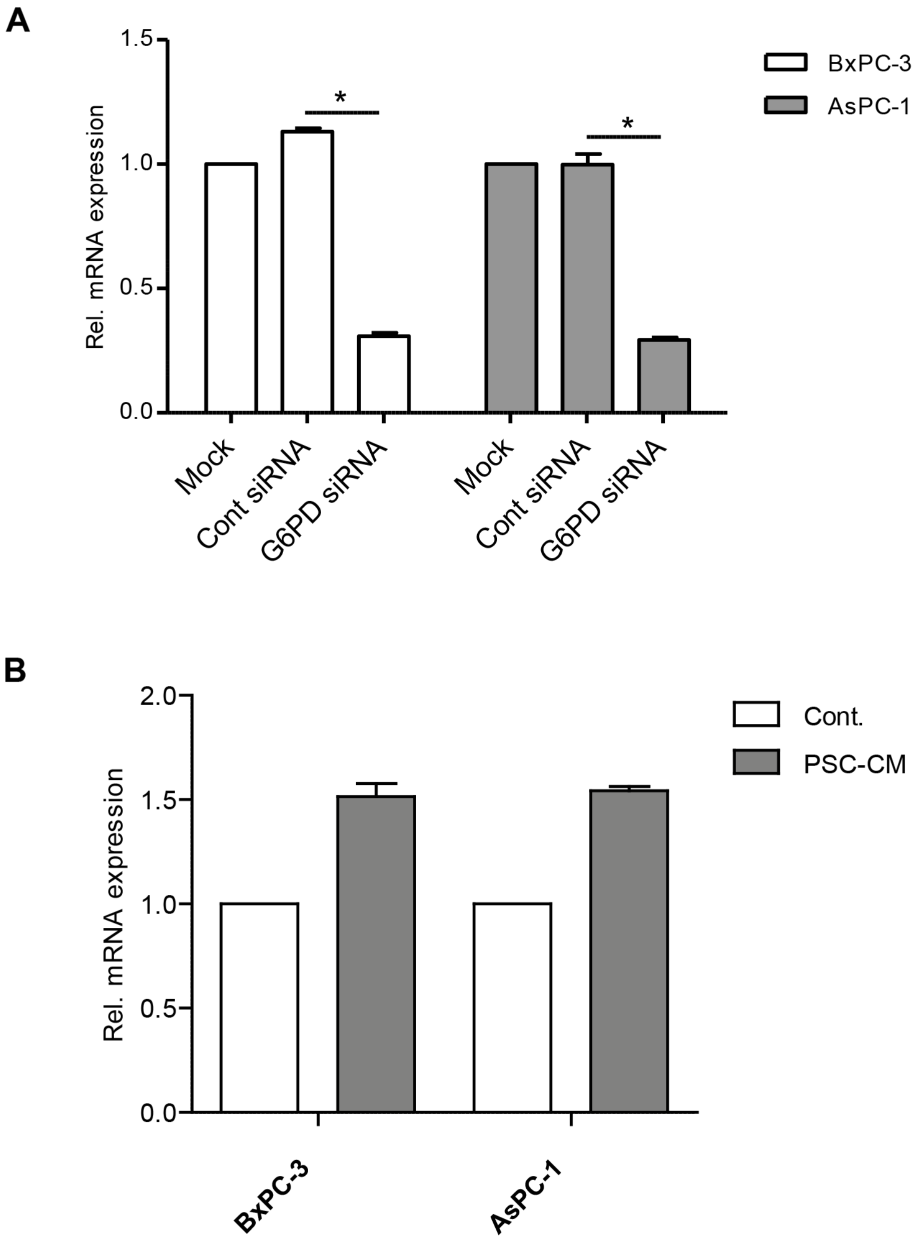

Supplementary Figure S4: G6PD gene expression after RNAi-mediated gene silencing. Upon transfection of PDAC cells with G6PD siRNA, control siRNA or transfection reagent alone for 48 h, the mRNA levels of G6PD was measured with qPCR **A.** Some cells were further treated with PSC-CM (1.0 µg/µl) for another 72 h, before analysis of G6PD expression **B.** Data are expressed as mean ± SD.*, P<0.05 G6PD siRNA versus control siRNA.

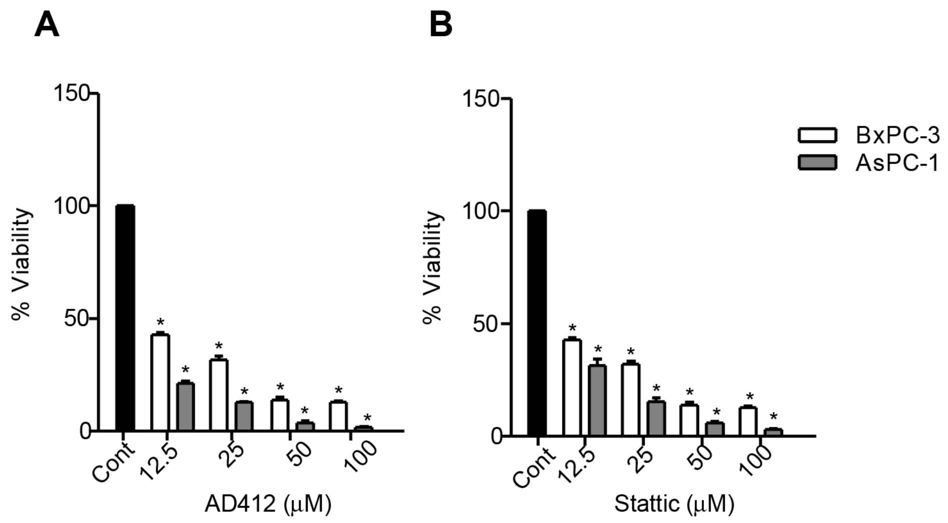

Supplementary Figure S5: Inhibition of JAK3 and Stat3 signaling abrogated PSC-CM-mediated PDAC cell proliferation. A, B. Cells were treated with AD412 and Stattic in the presence of PSC-CM (1.0 μg/μl) for 72 h. MTT assay was used to determine the cell viability. All experiments were performed in triplicates, and data are expressed as mean ± SD. *, $P<0.05$ versus cells treated with PSC-CM.

Supplementary Table S1: List of primer pairs used for qRT-PCR

Gene	Forward primer (5′-3′)	Reverse primer (5′-3′)
GAPDH	CCCATCACCATCTTCCAGGA	GTTGTCATGGATGACCTTGGC
Keap1	CAGATTGGCTGTGTGGAGTT	GCTGTTCGCAGTCGTACTTG
Nrf2	GAGAGCCCAGTCTTCATTGC	TTGGCTTCTGGACTTGGAAC
HMOX1	GGTAAGAACCAGGTCCGTCA	GGGCACTAACTCCCGTTACA
CAT	CATGCTGAATGAGGAACAGA	TTGTCCAGAAGAGCCTGGAT
AKR1c1	ATTCCCATCGACCAGAGTTG	TTTGGGATCACTTCCTCACC
NQO1	GAAGAGCACTGATCGTACTGGC	GGATACTGAAAGTTCGCAGGG
SOD1	GGTGGGCCAAAGGATGAAGAG	CCACAAGCCAAACGACTTCC
SOD2	GACAAACCTCAGCCCTAACG	CTGATTTGGACAAGCAGCAA
SOD3	ATGCTGGCGCTACTGTGTTC	CTCCGCCGAGTCAGAGTTG
G6PD	ACCGCATCGACCACTACCT	TGGGGCCGAAGATCCTGTT
PGD	ATGGCCCAAGCTGACATCG	AAAGCCGTGGTCATTCATGTT
TKT	CCTACACCGGCAAATACTTCG	GCCTCCCATACAGAGCCCT
TALDO1	CTCACCCGTGAAGCGTCAG	GTTGGTGGTAGCATCCTGGG
PPAT	AATTGTCAGCCCTTCGTTGTT	CCTTAATCGAGCAGCATTTACCA
MTHFD2	AGGACGAATGTGTTTGGATCAG	GGAATGCCAGTTCGCTTGATTA
ME1	GAGTGCTGACATCTGACATTGA	TTGGCTTCCGAAACACCAAAC
IDH1	AGAAGCATAATGTTGGCGTCA	CGTATGGTGCCATTTGGTGATT
GCLC	GGCGATGAGGTGGAATACAT	GGGTAGGATGGTTTGGGTTT
GCLM	CATTTACAGCCTTACTGGGAGG	ATGCAGTCAAATCTGGTGGCA
